# A Three-Dimensional Collagen-Elastin Scaffold for Heart Valve Tissue Engineering

**DOI:** 10.3390/bioengineering5030069

**Published:** 2018-08-28

**Authors:** Xinmei Wang, Mir S. Ali, Carla M. R. Lacerda

**Affiliations:** Department of Chemical Engineering, Texas Tech University, Lubbock, TX 79409, USA; xinmei.wang@ttu.edu (X.W.); mir.ali@ttu.edu (M.S.A.)

**Keywords:** collagen-elastin construct, gel scaffolds, heart valve regeneration, valvular interstitial cell phenotypes

## Abstract

Since most of the body’s extracellular matrix (ECM) is composed of collagen and elastin, we believe the choice of these materials is key for the future and promise of tissue engineering. Once it is known how elastin content of ECM guides cellular behavior (in 2D or 3D), one will be able to harness the power of collagen-elastin microenvironments to design and engineer stimuli-responsive tissues. Moreover, the implementation of such matrices to promote endothelial-mesenchymal transition of primary endothelial cells constitutes a powerful tool to engineer 3D tissues. Here, we design a 3D collagen-elastin scaffold to mimic the native ECM of heart valves, by providing the strength of collagen layers, as well as elasticity. Valve interstitial cells (VICs) were encapsulated in the collagen-elastin hydrogels and valve endothelial cells (VECs) cultured onto the surface to create an in vitro 3D VEC-VIC co-culture. Over a seven-day period, VICs had stable expression levels of integrin β1 and F-actin and continuously proliferated, while cell morphology changed to more elongated. VECs maintained endothelial phenotype up to day five, as indicated by low expression of F-actin and integrin β1, while transformed VECs accounted for less than 7% of the total VECs in culture. On day seven, over 20% VECs were transformed to mesenchymal phenotype, indicated by increased actin filaments and higher expression of integrin β1. These findings demonstrate that our 3D collagen-elastin scaffolds provided a novel tool to study cell-cell or cell-matrix interactions in vitro, promoting advances in the current knowledge of valvular endothelial cell mesenchymal transition.

## 1. Introduction

Heart disease is the most challenging and severe health problem worldwide, accounting for one in seven deaths in the US [1]. According to the most updated report from the American Heart Association, the prevalence of valvular heart disease is 2.5% of the total population in the US, a number which steadily increases with age [1]. The prevalence of valvular heart disease may double in the next 20 years and triple in the next 40 years based on current trends [2]. Due to the lack of effective medical therapies, surgical repair and replacement are commonly required as the primary treatment of valvular heart disease [3]. Approximately 100,000 valve replacement surgeries are conducted in the US each year [4,5]. However, the feasibility of heart valve replacement is restricted by many factors, such as limited heart valve tissues [6]. In this case, tissue engineering constitutes a promising technique to generate heart valves tissue constructs that mimic or act to repair the microenvironment and function of native heart valves. 

Heart valves possess a trilaminar architecture. The three layers are fibrosa, spongiosa, and ventricularis in semilunar valves (SVs) or atrialis in atrioventricular valves (AVs) [3]. These three layers are dominated by collagen, proteoglycans, and elastin, respectively, each with variable biomechanical properties [3]. Collagen fibers are circumferentially aligned in fibrosa and provide mechanical rigidity and strength, while elastin fibers are radially aligned in ventricularis/atrialis to support tissue movement due to their elasticity [7]. Proteoglycans located in the middle layer of extracellular matrix (ECM) provide tissue lubrication and cushioning [8]. This trilaminar architecture has yet to be modeled in vitro and the knowledge of its molecular contributions to valvular cells is lacking. Only by developing a 3D valvular cell construct in a biochemically-suitable environment (composed of collagen, elastin, and proteoglycans), one will be able to determine the most important variables to be considered in the design and engineering of novel products for heart valve replacement and repair.

Two major cell populations are present in heart valves. They are endothelial cells lining both inflow and outflow surfaces and interstitial cells distributed throughout the inner space of heart valves. Valvular endothelial cells (VECs) are sensitive to mechanical forces such as in vivo hemodynamic conditions, and they are capable of transducing mechanical signals to biological signals by altering morphologies and interacting with interstitial cells [7,9]. Valvular interstitial cells (VICs) are a group of heterogeneous cells with different phenotypes that may transform into each other under pathological triggers [10]. In healthy heart valves, over 95% of VICs show a fibroblastic quiescent phenotype [10]. In valve remodeling, quiescent VICs are activated and transform into myofibroblasts with increased expression of protein markers, such as α-smooth muscle actin (α-SMA) [10]. In the specific case of valvular calcification, osteoblastic VICs are originated and cooperate with activated VICs for tissue remodeling [10]. Activated and osteoblastic VICs are capable of producing and secreting matrix metalloproteinases (MMPs) and tissue inhibitors of matrix metalloproteases (TIMPs) to alter the organization of ECM, which typically occurs during valvular degenerative diseases [3]. 

In addition to the transformation from quiescent VICs, activated VICs have recently been shown to originate from endothelial to mesenchymal transition (EndoMT) [11]. EndoMT has a dual role in regulating biological processes. It has positive effects during embryonic valvulogenesis and injury repair, and negative effects in a variety of diseases, such as cardiac fibrosis, cancer, and valvular degeneration [12,13,14]. Still, EndoMT constitutes a promising route to reconstruct tissues/patches to be applied in regenerative medicine. If progenitor VICs and endothelial progenitors are employed, EndoMT can harness their differentiation abilities, potentially leading to more successful constructs than those developed with mesenchymal stem cells from other adult tissues [15,16]. In EndoMT, cells transition from endothelial to mesenchymal phenotype by losing cell-cell contacts and endothelial cell markers such as CD31, while acquiring mesenchymal cell markers [16]. Subsequently, transformed cells invade the neighboring 3D environment and function as myofibroblasts to remodel the ECM [17]. 

Previous studies demonstrate that EndoMT can be triggered by mechanical forces [17,18]. The current literature on mechanically-triggered EndoMT encompasses a number of co-culture studies characterizing loss of endothelial and gain of mesenchymal characteristics, not necessarily evaluating 3D infiltration [15,19]. Mechanically-triggered EndoMT in vitro has been studied in the contexts of multiple fibrotic diseases [11,20], tissue regeneration [15], angiogenesis [21], and pulmonary hypertension [22,23], in addition to valvular disease [13]. It has only been recently proposed that EndoMT plays a role in the early stages of valvular heart disease [24], in addition to its well-established role in valvular development [25]. In a previously-designed 3D model, EndoMT was induced with the activation of inflammatory molecules, and modulated by shear stress in a pattern and magnitude-dependent manner [24]. In another mechanical stimulus study, EndoMT was successfully induced by cyclic strain in both low and high magnitudes, but mediated by different signaling pathways [18]. Desirable properties of a tissue-engineered heart valve include similar mechanical environment, similar chemical environment, durability, low inflammatory and antigenic potentials, ability to diffuse nutrients and waste, amenable to cell adhesion, migration, proliferation and differentiation as needed. Specifically to heart valves, it is important that constructs promote VIC quiescence for proper homeostatic control [26]. Here, we design a 3D hydrogel scaffold made of collagen and elastin to mimic the native ECM of heart valves and provide the strength of collagen layers as well as elastic fibers. VICs were encapsulated in the collagen-elastin hydrogels and VECs cultured onto the surface to create an in vitro 3D VEC-VIC co-culture. For both VECs and VICs, cell numbers were counted every other day up to seven days after seeding, and proliferation rates were calculated based on cell counts. EndoMT and cell migration were evaluated in a seven-day culture from immunofluorescence images. Ultimately, we expect this experimental design to lay the groundwork for testing of a construct made of autologous progenitor VICs and adult endothelial progenitors.

## 2. Materials and Methods

Preparation of 3D cellularized constructs: Primary porcine aortic valvular interstitial cells (PAVICs) and endothelial cells (PAVECs) from passage 3–5 were used in this study. Fresh porcine hearts were obtained from a local slaughterhouse (ethics approval not required) and transported to the lab on ice as described previously [27]. Porcine aortic valve cusps were cut and washed in sterile phosphate-buffered saline (1 × PBS, 137 mM sodium chloride, 4.3 mM sodium phosphate dibasic, 2.7 mM potassium chloride, and 1.46 mM potassium phosphate monobasic) with 2% amphotericin b/penicillin/streptomycin (Quality Biological, Gaithersburg, MD, USA) for at least 3 times before cell isolation. After thoroughly washing, aortic valves were incubated for 10 min in 600 U/mL collagenase (Sigma-Aldrich, St. Louis, MO, USA) solution, which was prepared in fresh cell culture medium (Dulbecco’s modified Eagle medium (DMEM) (Corning Life Sciences, Tewksbury, MA, USA), 10% fetal bovine serum (FBS) (Atlanta Biologicals, Flowery Branch, GA, USA), 1% amphotericin b/penicillin/streptomycin), at 37 °C under a humidified atmosphere and 5% CO_2_. PAVECs were removed using a sterile soft brush after 10 min of incubation and collected by centrifugation at 1100 rpm for 5 min. Harvested PAVECs were cultured in 12-well plates and maintained in heparin medium (50 U/mL heparin (Sigma-Aldrich, St. Louis, MO, USA) in DMEM, 10% FBS, and 1% amphotericin b/penicillin/streptomycin, filter-sterilized) at 37 °C under a humidified atmosphere and 5% CO_2_. After PAVEC removal, aortic valves underwent complete collagenase digestion overnight for PAVIC isolation. PAVICs were then cultured in complete cell culture medium (DMEM, 10% FBS, and 1% amphotericin b/penicillin/streptomycin) at 37 °C under a humidified atmosphere and 5% CO_2_. Upon reaching 80% confluence, cells were harvested using 0.125% trypsin solution (Sigma-Aldrich, St. Louis, MO, USA) for 3D collagen-elastin experiments.

**Figure 1:**
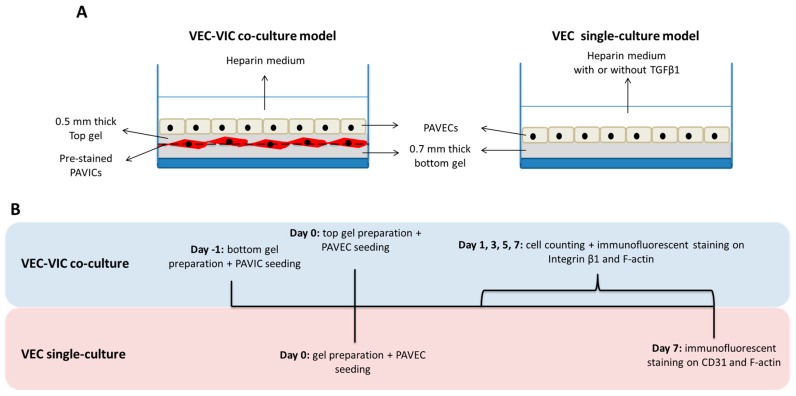
(**A**) Schematic of 3D valve endothelial cell (VEC)-valve interstitial cell (VIC) co-culture model and VEC single-culture model. (**B**) Experimental timeline of each 3D model.

Collagen-elastin gels were prepared with 50% bovine type I collagen solution (from a 6 mg/mL stock, Advanced Biomatrix, Carlsbad, CA, USA), 12% elastin solution made from sterile 5 mg/mL stock solution (Sigma-Aldrich, St. Louis, MO, USA), 10% PBS (10×), and the balance completed with equal parts of DMEM and FBS. Final concentrations of collagen and elastin in the gel were 3 mg/mL (*w*/*v*) and 0.6 mg/mL (*w*/*v*). After adjusting the pH with 1 M sterile sodium hydroxide to 7.5, the solution was transferred to 37 °C under a humidified atmosphere and 5% CO_2_ and allowed to gel for 1 h. Both VEC-VIC co-culture and VEC single-culture models were created using these gels, as shown in Figure 1A. The timeline of the entire culture period of each model is described in Figure 1B. 

The 3D cell-embedded VEC-VIC co-cultures were created by assembling two collagen-elastin hydrogel layers with a PAVIC layer in between and PAVECs on top after construct stabilization (Figure 1A). First, a 0.7 mm thick bottom gel was created at 37 °C and 5% CO_2_. Before seeding onto the bottom gel, PAVICs were had their membranes tagged with the lipophilic carbocyanine DiD (Vybrant Multicolor Cell-Labeling Kit, Life Technologies, Carlsbad, CA, USA) following the manufacturer’s protocol. This lipophilic fluorophore penetrates the cell membranes and are passed down to daughter cells after many cycles of cell division. Briefly, 10^6^ suspended cells were incubated in 1 mL serum-free medium (DMEM, 10% serum replacement (Sigma-Aldrich, St. Louis, MO, USA), 1% amphotericin b/penicillin/streptomycin) containing 0.5% DiD solution (far-red emission) for 20 min at 37 °C and 5% CO_2_. Then, 1.25 × 10^5^ pre-labelled PAVICs were seeded onto the surface of each bottom gel and maintained in complete cell culture medium. After overnight attachment of PAVICs, a new liquid layer was placed onto the top of PAVICs and allowed to gel to create a 0.5 mm thick top gel at 37 °C and 5% CO_2_. Once the top gel was successfully formed, 10^5^ PAVECs were seeded on top to complete 3D cell-hydrogel constructs. 

For co-culture controls, VEC single-cultures were created by seeding VECs on 0.7 mm thick collagen-elastin gels. In single cultures, VECs were maintained in heparin medium only, negative control, and for EndoMT stimulation (positive control) heparin medium supplemented with 10 ng/mL recombinant human transforming growth factor beta 1 (TGFβ1, R&D Systems, Minneapolis, MN, USA) for 7 days (Figure 1A).

Assessment of EndoMT: Cells were fixed in 2% formaldehyde (Thermo Fisher Scientific, Waltham, MA, USA) for 40 min, followed by permeabilization with 0.1% Igepal in MES buffer (Sigma-Aldrich, St. Louis, MO, USA) (10 mM 2-(N-morpholino) ethanesulfonic acid, 10 mM sodium chloride, 1.5 mM magnesium chloride, 10% glycerol, 100 KIU aprotinin, pH = 6.2) for 10 min and blocking with 1% goat serum (MP Biomedicals, Santa Ana, CA, USA) in MES buffer for 40 min before antibody binding. In VEC-VIC co-cultures, integrin β1 was labeled with a mouse monoclonal primary antibody (Thermo Scientific, Waltham, MA, USA) with a final concentration of 2.5 µg/mL, while in VEC single-culture model, CD31 was labeled with a mouse monoclonal primary antibody (Sigma-Aldrich, St. Louis, MO, USA) with a final concentration of 2 µg/mL. After overnight incubation with integrin β1 or CD31 primary antibody, cells were stained by DyLight 488 conjugated goat anti-mouse IgG secondary antibody (Thermo Scientific, Waltham, MA, USA). Actin cytoskeleton and nuclei were stained with 1 U/mL rhodamine-phalloidin (Cytoskeleton, Denver, CO, USA) for 45 min and 1 μg/mL 4′,6-diamidino-2-phenylindole (DAPI) (Molecular Probes, Eugene, OR, USA) for 10 min. Fluorescent images were acquired using a Leica microsystem (AF6000, imaging software Leica Application Suite X, Leica Microsystems, Wetzlar, Germany). 

In VEC-VIC co-cultures, PAVECs and PAVICs were separately counted on day 1, 3, 5, and 7 (Figure 1B) from at least 25 DAPI-labeled 200× fluorescent images. Subsequently, proliferation rates of PAVICs and PAVECs of every 2 days were determined by counting average cell numbers. In addition to counting total cell numbers, in VEC-VIC co-cultures, the ratios of transformed VECs over total VECs in day 1, 3, 5, and 7 cultures were measured from at least 25 fluorescent images (200×), where F-actin was labeled by rhodamin-phalloidin and nuclei were labeled by DAPI. Cell migration caused by EndoMT from day 7 cultures was investigated from a series of z-stack images with 20 μm z-steps. EndoMT occurring in VEC single-culture model with or without TGFβ1 was evaluated according to changes in expression of endothelial and mesenchymal markers, as indicated in CD31 and F-actin labeled fluorescent images. Data were presented as mean ± standard error. Normal distribution of each data group was tested by one-sample Kolmogorov-Smirnov test. Subsequently, statistically significant differences were assessed by one-way ANOVA and multiple comparison tests, and *p*-values < 0.05 were considered significant.

## 3. Results

**Figure 2:**
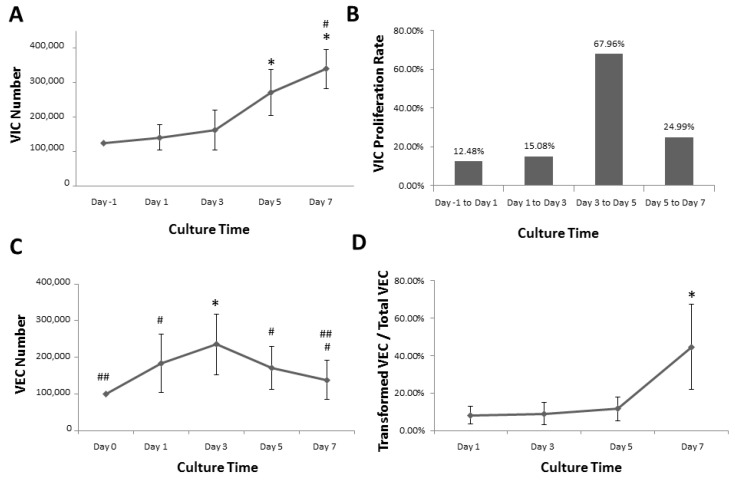
(**A**) VIC counts in 3D VEC-VIC co-culture model. * *p* < 0.05, comparing with seeding, day 1, and day 3; ^#^ *p* < 0.05, comparing with day 5; (**B**) VIC proliferation rates in 3D VEC-VIC co-culture model. (**C**) VEC counts in 3D VEC-VIC co-culture model. * *p* < 0.05, comparing with seeding, day 1, day 5, and day 7; ^#^ *p* < 0.05, comparing with seeding; “^##^” indicating day 7 not significantly different from seeding; (**D**) Ratios of transformed VECs over total VECs. * *p* < 0.05, comparing with day 1, day 3, or day 5.

VIC behavior in 3D VEC-VIC co-culture: VICs were used as a biological glue between two hydrogels and allowed to grow for 7 days. The total VIC number in each 3D hydrogel structure was counted from nuclei-labelled 200× fluorescent images every other day (days 1, 3, 5, and 7) after assembling the complete co-culture. VICs proliferated up to 7 days, as indicated in Figure 2A. The initial seed count was 3.4 × 10^4^ cells/cm^2^, and that number of VICs doubled by day 5. Statistical tests determined that the cell counts from days 5 and 7 were significantly higher than those from day 1 and 3. Although the number of VICs was not significantly different from day 1 to day 3, the cell number from day 3 was notably increased by comparing with the initial seed count. Proliferation rates were determined by taking the ratio of cell counts on specific days over the previous day. These data indicated that, in the last two days of the culture period, there was a great increase in cell number, with a proliferation rate of approximately 25% (Figure 2A,B). VICs proliferated slowly until day 3, with a relatively consistent proliferation rate every 2 days. The proliferation rates were between 12% and 15% in the first few days in culture (Figure 2B). From day 3 to day 5, the proliferation rate was increased to 68%, followed by a decrease from day 5 to day 7. 

**Figure 3:**
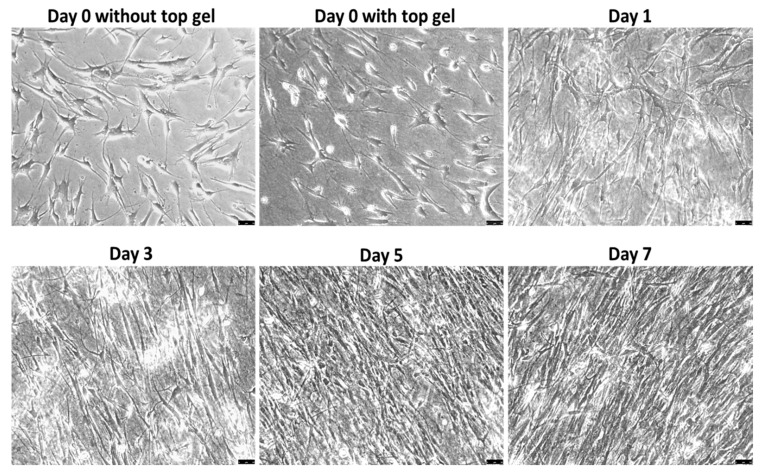
Phase-contrast images of VICs showing cell elongation and alignment with time. Scale bar: 50 μm.

**Figure 4:**
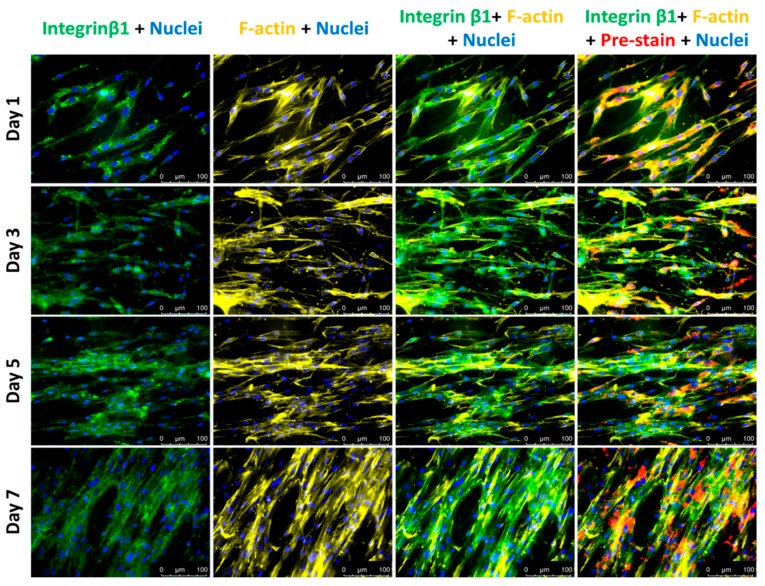
Fluorescent images of VICs in middle layer. Integrin β1—Green; F-actin—Yellow; Nuclei—Blue; Cell membrane—Red. Scale bar: 100 μm.

Culture confluence reached approximately 70% by day 5 and further increased to over 90% on day 7, as shown in Figure 3. Additionally, more cells aligned in highly confluent cultures. On later days in culture, cells aligned in parallel with each other, along their long axis, potentially due to collagen fiber orientation (Figure 3). This behavior is not commonly observed in other cultures. Before the observation of cell alignment on day 3, cell elongation happened one day after assembling the 3D co-culture and this elongated cell morphology was maintained with increased culture confluence and cell alignment. The morphological changes in cell length and alignment were also observed in F-actin-labeled fluorescent images, as shown in Figure 4. The expression levels of integrin β1 and F-actin remained the same throughout the entire culture period, even though there seems to be a small increase in the number of actin fibers per cell in day 7 cultures. 

VEC behavior in 3D VEC-VIC co-culture: VECs were cultured on the top of stacked 3D gels on day 0 with the initial seeding number of 2.7 × 10^4^ cells/cm^2^. The total VEC number on each 3.7 cm^2^ gel surface was counted from nuclei-labelled 200× fluorescent images every other day (days 1, 3, 5, and 7) on the highest focal plane of the gel after complete co-culture assembly. Unlike VICs, the number of VECs increased up to day 3, followed by a decrease in the last 4 days (Figure 2C). This observation of lower cell counts could be linked to three possible outcomes. First, detachment/loss of endothelial cells from the surface due to culture conditions, resulting in an apparent slower growth rate. Second, an actual decrease in cell growth rate coupled with cell detachment. Finally, endothelial cells may appear less abundant due to mesenchymal transformation. 

**Figure 5:**
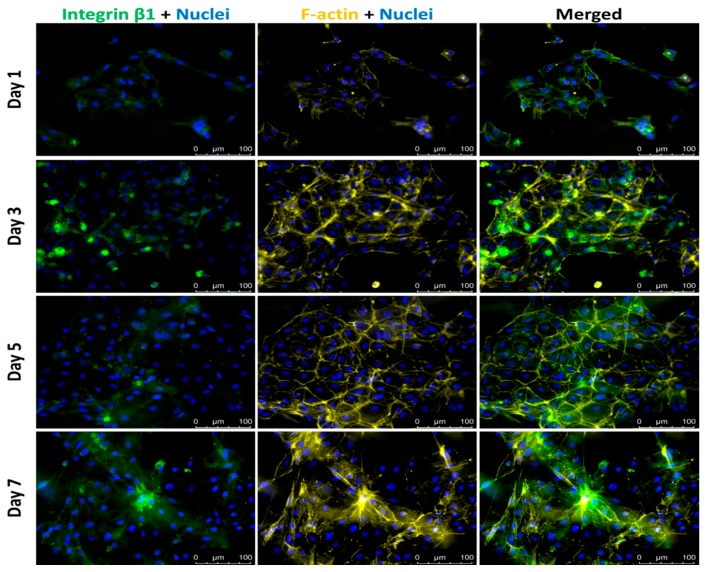
Fluorescent images of VECs on top layer of the VEC-VIC co-culture model. Integrin β1—Green; F-actin—Yellow; Nuclei—Blue. Scale bar: 100 μm.

The ratio of transformed VECs over total VECs was calculated from the number of cells with positive F-actin expression over the number of cells with negative F-actin expression on the top focal plane, considering that DiD-labeled VIC would be distinct from transformed VEC, in case they migrated to the top focal plane. As shown in Figure 2D, the ratios from cultures on days 1, 3, and 5 remained below 7%, while it sharply increased to over 20% on day 7. In day 7 cultures, migrated VECs were counted from nuclei-labelled z-stack images acquired from 15 distinct regions (Supplemental Video). On average, 8% of VECs migrated into the gel, a calculation based on the number of DiD-negative cells located in the gel interstitial space between the center and top focal planes originally populated with cells, VIC and VEC, respectively. In addition, VECs largely maintained their endothelial phenotype on the co-culture surface until day 5, past which accelerated EndoMT occurred. While VECs maintained endothelial phenotype, there was low expression of integrin β1 and F-actin, as indicated by day 1 fluorescent images in Figure 5. In day 3 and day 5 cultures, only a few cells expressed integrin β1, while VECs still kept cell-cell contacts with expression of only cortical F-actin. In the last day of culture, some cells produced more actin filaments, associated with increased expression of integrin β1. The increase in expression levels of mesenchymal markers indicates cells underwent EndoMT. 

**Figure 6:**
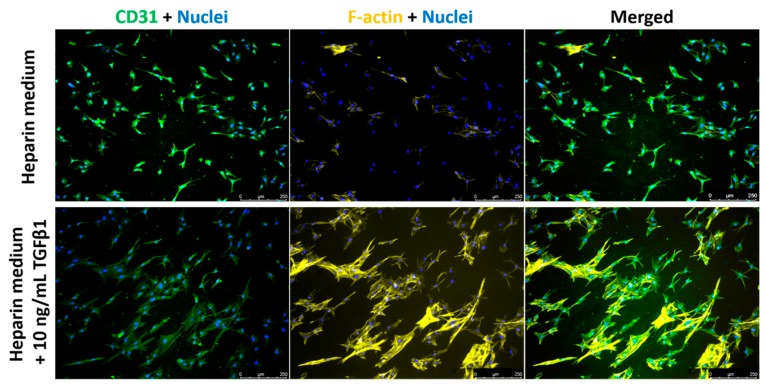
Fluorescent images of VECs in VEC single-culture model. CD31—Green; F-actin—Yellow; Nuclei—Blue. Scale bar: 250 μm.

VEC behavior in single culture: On a separate experiment, we employed a VEC pure culture model, where VECs were seeded onto the top surface of collagen-elastin gels and cultured for 7 days. The effect of TGFβ1 on EndoMT was investigated in this single cell model to assess levels of phenotype markers (loss of CD31 and increase in F-actin, non-cortical) in TGFβ1-supplemented cultures (Figure 6). After 7 days in culture, untreated VECs maintained their original CD31-positive phenotype, while the expression of F-actin was undetectable in the majority of the cells (Figure 6). However, in TGFβ1-supplemented cultures, cells showed more prominent actin filaments with reduced expression of CD31, indicating the occurrence of EndoMT (Figure 6). Along with the differential expression of protein markers, VECs cultured with heparin medium sustained the cobblestone endothelial morphology. With the presence of TGFβ1, VECs were elongated to more spindle-shaped morphology. However, VEC did not easily migrate into the gel matrix.

## 4. Discussion

Previous studies of VEC-VIC co-cultures, without 3D organization constraints, demonstrated that VECs reduced the activation of VICs by reducing the expression of myofibroblastic markers and stabilizing matrix synthesis and degradation in 3D [28,29,30]. In turn, VECs did not undergo EndoMT in the presence of VICs [30]. In the currently studied VEC-VIC co-cultures, the number of VECs was reduced after day 3, potentially due to cell losses or the inhibitory effects of elastin and collagen on endothelial cell proliferation, previously reported for vascular endothelial cells [31]. Endothelial cell phenotype was maintained up to 5 days, while VICs proliferated for over one week. It is also interesting to appreciate that VICs were spontaneously-aligned in static cultures, which might be caused by highly oriented collagen fibers in the hydrogel matrix [32]. 

The fibrosa layer mainly supports structural integrity of heart valves due to abundant collagen fibers [33,34]. Additionally, collagen was widely used as a scaffold due to its low antigenicity and biodegradability [35]. Here, we used collagen supplemented with elastin to fabricate gel constructs to confer necessary tissue stiffness and elasticity for VIC quiescence, as reported by many researchers in the field of valvular cell mechanotransduction [28,36,37]. A construct of this kind is only a good model of valvular physiology if cells are quiescent, thus a soft material such as our collagen-elastin gels is an important requirement. In this study, the assembled 3D collagen-elastin scaffolds possessed an environment bio-chemically similar to those of native heart valves [38], and capable of supporting cell attachment, migration, differentiation, and proliferation. Moreover, this 3D model has tunable biomechanical properties by varying the ratio of collagen and elastin. More importantly, to our knowledge, this is the first study reporting 3D in vitro models of valvular physiology, using collagen-elastin hydrogels without chemical crosslinker and assembled by temperature triggered gelation [39,40], which simplified and improved previous procedures and reduced cell toxicity [41,42]. In VEC-VIC co-culture, the top and bottom hydrogels were directly stacked and they were strongly bound due to the adhesive forces between encapsulated VICs and surrounding collagen-elastin matrix, as indicated by the strong expression of integrin β1 in VICs. The integrity of 3D constructs was maintained during the entire experimental period, making these constructs amenable to the development of multilayer designs with multiple types of ECM components and cells embedded. 

Future experiments using this in vitro model plan to address important limitations of this study, such as tunable composition differences in the mechanical environment, and, most importantly, the lack of pulsatile stimulation, both currently being investigated in our laboratory. In addition, we plan to test biological cues to promote or reverse EndoMT. For example, it has been demonstrated that BMP7 functions as a TGFβ1 antagonist, promoting mesenchymal to endothelial transition [43]. However, the understanding of how BMP7, TGFβ1, and co-cultured cells interplay to regulate EndoMT is lacking. This research will provide a better understanding of EndoMT control, which will contribute to the development of engineered tissues, and novel therapeutic strategies for valvular heart disease. In addition, different types and magnitudes of mechanical forces are currently being applied to collagen-elastin hydrogel constructs to investigate dynamic mechanical effects on valvular cell interactions and explore signaling mechanisms regulating mechanically induced valvular cell transformation in 3D models. 

## 5. Conclusions

3D collagen-elastin hydrogel constructs in this study were designed to mimic the biochemical microenvironment of native tissues with VIC and VEC in co-culture. In an experimental period of 7 days, VICs continuously proliferated and the cell number was doubled on day 5, while cell morphology changed to become more elongated and aligned with time. VICs had stable expression levels of integrin β1 and F-actin during the entire culture period. While VECs seemed highly proliferative on the gel surface, cell losses were observed. The expression of integrin β1 remained low in VECs, as expected. VECs maintained endothelial phenotype up to day 5, as indicated by low expression of F-actin, while transformed VECs accounted for less than 7% of the total VECs in culture. On day 7, over 20% VECs were transformed to mesenchymal phenotype, indicated by increased actin filaments and higher expression of integrin β1. In addition, EndoMT was observed from VEC single-cultures due to TGFβ1 treatment. Based on these findings, our 3D collagen-elastin scaffolds provided a novel tool to study cell-cell or cell-matrix interactions in vitro, promoting advances in heart valve tissue engineering. 

## Supplementary Materials

The following are available online at http://www.mdpi.com/2306-5354/5/3/69/s1, Video S1.
